# Discussion paper: implications for the further development of the successfully in emergency medicine implemented AUD_2_IT-algorithm

**DOI:** 10.3389/fdgth.2024.1249454

**Published:** 2024-04-04

**Authors:** Christopher Przestrzelski, Antonina Jakob, Clemens Jakob, Felix R. Hoffmann

**Affiliations:** ^1^ Department of Traumasurgery and Orthopedics, Klinikum Bogenhausen, Munich, Germany; ^2^ Surgical Management LMU Munich University Hospital, Munich, Germany; ^3^ Strategy & Market Research, Generali Deutschland AG, Munich, Germany; ^4^ Department of Health Economics, APOLLON University of Applied Sciences, Bremen, Germany

**Keywords:** handover, emergency medicine, process management, interoperability (IoP), data

## Abstract

The AUD_2_IT-algorithm is a tool to structure the data, which is collected during an emergency treatment. The goal is on the one hand to structure the documentation of the data and on the other hand to give a standardised data structure for the report during handover of an emergency patient. AUD_2_IT-algorithm was developed to provide residents a documentation aid, which helps to structure the medical reports without getting lost in unimportant details or forgetting important information. The sequence of anamnesis, clinical examination, considering a differential diagnosis, technical diagnostics, interpretation and therapy is rather an academic classification than a description of the real workflow. In a real setting, most of these steps take place simultaneously. Therefore, the application of the AUD_2_IT-algorithm should also be carried out according to the real processes. A big advantage of the AUD_2_IT-algorithm is that it can be used as a structure for the entire treatment process and also is entirely usable as a handover protocol within this process to make sure, that the existing state of knowledge is ensured at each point of a team-timeout. PR-E-(AUD_2_IT)-algorithm makes it possible to document a treatment process that, in principle, does not have to be limited to the field of emergency medicine. Also, in the outpatient treatment the PR-E-(AUD_2_IT)-algorithm could be used and further developed. One example could be the preparation and allocation of needed resources at the general practitioner. The algorithm is a standardised tool that can be used by healthcare professionals of any level of training. It gives the user a sense of security in their daily work.

## Introduction

In order to ensure that medical data management is of benefit to patients, it is necessary for it to be collected in a standardized manner and documented in an interoperable manner. A distinction can be made between different levels of interoperability. Structural interoperability means the ability to transfer user data from one system to another. Syntactic interoperability describes the ability to identify individual information units and data structures in the transmitted user data and to extract them for the purpose of further processing. Semantic interoperability describes the ability to interpret the extracted information units semantically correctly. Finally, the organizational interoperability describes cross-organisational coordinated workflows and processes (1).

The AUD_2_IT-algorithm is a tool to structure the data, which is collected during an emergency treatment. The two main goals are first to structure the documentation of the data and second to give a standardised data structure for the report during handover of an emergency patient. Accordingly, the AUD_2_IT-algorithm serves to ensure organizational interoperability in emergency medicine (2, 3).

The AUD_2_IT-algorithm was once developed to structure treatment reports in the emergency room in a uniform manner across all disciplines. In practice, it became apparent, that not only the quality of the reports was improved, but the scheme was also ideal for handing over patients. After a while, the AUD_2_IT-algorithm was taken up by professional societies, changed in some points, renamed to (PR_E-)AUD^2^IT-algorithm and as such included in the curriculum of emergency room-trainings.

Today the (PR_E-)AUD^2^IT-algorithm is deeply introduced in training curriculums of emergency medicine (4) and is recommended to be used in emergency departments for treatment of critical ill, non-traumatic patients (5). But in some points, there is no uniform interpretation of the application of the scheme. This discussion paper aims to reflect the existing publications and user experiences regarding the AUD_2_IT-algorithm, will discuss the controversial points and make recommendations for the implementation.

### AUD_2_IT-algorithm and (Pr_E-)AUD^2^IT-algorithm in emergency care

The idea behind the development of the AUD_2_IT-algorithm was to provide residents a documentation aid, which helps to structure the medical reports without getting lost in unimportant details or losing important information. Therefore, the AUD_2_IT-algorithm maps the entire emergency treatment and brings together the data in clusters, which are documented chronologically. A pleasant side effect is, that the AUD_2_IT-algorithm can replace or include many of the numerous emergency medical action schemes such as SBAR, SAMPLER or cABCDE, thus only one single action scheme must be learned (2, 3).

The treatment process in a resuscitation room commences well before the patient even arrives. The resuscitation team is actively engaged in preparing for the patient's arrival and the entire process is carefully orchestrated to ensure the best possible outcome. So, there was a need to expand the AUD_2_IT-algorithm to include the phase from the alarm to the arrival of the patient in the resuscitation room. The (PR_E-)AUD^2^IT-algorithm was developed (6). Table 1 compares and describes the two algorithms.

**Table 1:** Comparison of AUD_2_IT-algorithm and (PR_E-)AUD^2^IT-algorithm.

Item	AUD_2_IT-Algorithm (German-language original terms)	AUD_2_IT-Algorithm (English translation)	AUD_2_IT-Algorithm (Action for each Item)	(PR_E-)AUD^2^IT-Algorithm (German-language original terms)	(PR_E-)AUD^2^IT-Algorithm (English translation)	(PR_E-)AUD^2^IT-Algorithm (Action for each Item)
P	/	/	/	Präparation	Preparation	Team Alerted. Check if equipment is available and functional. Check personal protective equipment.
R	/	/	/	Ressourcen	Ressources	Check the availability of all resources (radiology, laboratory, blood bank, IMC/ITS, endoscopy, catheter laboratory).
_	/	/	/	_Pause (Vorbereitung/Übergabe)	_Pause (preparation/patient handover)	Team-Time-Out. Handover by ambulance services.
E	/	/	/	Erstversorgung	Primary survey	Treatment according to ABCDE-scheme. Evaluate, if patient is stable or not. Determine main problem.
–	/	/	/	Team-Time-Out	Team-Time-Out	Team-Time-Out. Planning the next steps.
A	Anamnese	Anamnesis	Current symptoms Development over time Pre-existing illnesses Current Medication Allergies	Anamnese	Anamnesis	SAMPLER, OPQRST
U	Klinische Untersuchung	Clinical Examination	Clinical examination using the cABCDE-Algorithm.	Untersuchung	Examination	Clinical examination using the ABCDE-Algorithm including basic apparative diagnostics like ecg or sonography.
D	Differentialdiagnosen	Differential Diagnosis	Which suspected diagnoses are probable based on the anamnesis and clinical examination?	Differentialdiagnosen	Differential Diagnosis	Which suspected diagnoses are probable based on the anamnesis and clinical examination?
D	Apparative Diagnostik	Apparative Diagnostic	Carrying out further apparative diagnostic to rule out suspected diagnoses.	Diagnostik	Diagnostic	Carrying out further apparative diagnostic to rule out suspected diagnoses.
I	Interpretation	Interpretation	Collect and interpret all findings and make a diagnosis.	Interpretation	Interpretation	Collect and interpret all findings and make a diagnosis.
T	Therapie	Therapy	Create an individual treatment plan. Provide immediate emergency treatment as needed. Referral of the patient to where the treatment is continued.	To Do	To Do	Further Diagnostic, Therapy, planning the Debriefing.

### Considerations about how to improve the implementation of the (Pr_E-)AUD^2^IT-algorithm in emergency care

While the (PR_E-)-section represents a chronological period of time in preparation for care for the emergency patient, the period during the AUD^2^IT-section is more complicated. The sequence of anamnesis, clinical examination, considering a differential diagnosis, technical diagnostics, interpretation and therapy is rather an academic classification then a description of the real workflow. In a real setting, most of these steps take place simultaneously. Therefore, the application of the AUD_2_IT-algorithm should also be carried out according to the real processes.

**Figure 1:**
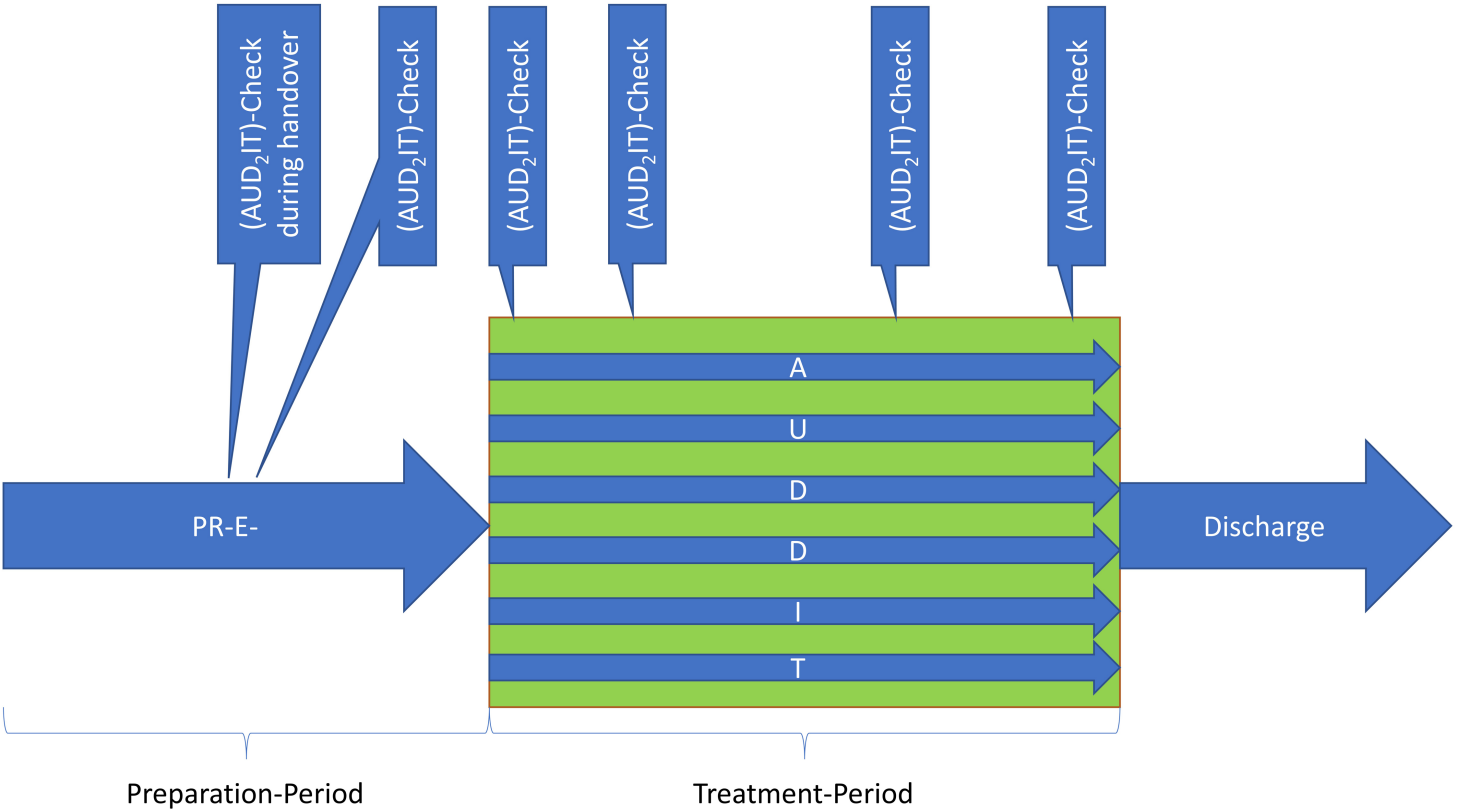
Using AUD_2_IT-algorithm as a structure for the entire treatment process and also to summarize the existing state of knowledge at each point of a team-timeout.

Bernhard et al. say that handover is an important point within an emergency treatment and gives advice, which general conditions should be ensured during the handover-process (5). But there is lack of information about the contents and the structure of the handover. As a result, healthcare professionals may resort to employing pre-existing handover protocols; however, these protocols are frequently incomplete and may not adequately address all necessary actions. That is not necessary as the AUD_2_IT-algorithm can be used instead and therewith can replace most of the existing action schemes. The clue is, that on the one hand the AUD_2_IT-algorithm can be used as a structure for the entire treatment process and also is entirely used within this process to summarize the existing state of knowledge at each point of a team-timeout (Figure 1). It is not necessary to repeat every information at every time when the team has not changed. It is enough just to summarize changes or new findings since the last AUD_2_IT-check.

In this way, the AUD_2_IT-algorithm gets a new role and can not only be used for treatment planning and documentation, but also for all handovers.

The “U” changed during the time. While it described in the initial AUD_2_IT-algorithm the clinical examination, in (PR_E-)AUD^2^IT-algorithm it also includes an ecg or sonography (2, 3, 6). This should be critically questioned since these apparatus diagnostic are also the consequence of the anamnesis and clinical examination. In an emergency treatment, several steps take place at the same time, so it would make more sense to assign each person with a different task and thus carry out all measures according to the AUD_2_IT-algorithm at the same time.

The treatment documentation should be done according to the AUD_2_IT-algorithm by using international standards for interoperability like FHIR or HL7.

### The nomenclature

Let us discuss two aspects of the nomenclature.

In the original version of the AUD_2_IT-algorithm, the ″2″ is subscript to make clear that it stands for two D's. The ″2″ in the initial publication of the (PR_E-)AUD^2^IT-algorithm is superscript, giving it meaning and suggesting, that the “D” is being squared. We recommend to go back to the original variant with a subscript ″2″, as it is also the case in other algorithms like the CHA_2_DS_2_-VASc-Score (7).

We would like to propose a recommendation on the use of brackets and hyphen. To do justice to the fact that (PR_E-) describes a chronological process while AUD_2_IT should be used repeatedly during an ongoing treatment process, we recommend to put the AUD_2_IT in brackets. The underscore and the hyphen both represent a team time out; to avoid confusion, we recommend using in both cases the same special character. Consequently, we recommend to use the notation PR-E-(AUD_2_IT) for the complete algorithm.

### Can the Pr-E-(AUD_2_IT)-algorithm be used in non-emergency medical fields?

The PR-E-(AUD_2_IT)-algorithm makes it possible to document a treatment process that, in principle, does not have to be limited to the field of emergency medicine. After treatment in the emergency department, often follows a transition to inpatient care. The fact, that the illness occurred acutely, does not play a significant role in the handover and documentation. When the patient moves from the emergency department to another unit, data is also transferred. This often results in interface breaks, which can be countered by a uniform design of the data infrastructure. Why should it not be possible for the documentation of a treatment to be continued in the same way even after the transfer to another unit?

In principle, all inpatient and outpatient treatments at case level could be documented with the AUD_2_IT-algorithm. The result would be a clear standardization of data infrastructures, which is for example reflected in uniform treatment reports and user interfaces of IT applications. Further studies should evaluate whether these considerations can also be proven in practice.

Also, in the outpatient treatment the PR-E-(AUD_2_IT)-algorithm could be used and further developed. One example could be the preparation and allocation of needed resources at the general practitioner. The algorithm is a standard tool that can be used by medical personnel of any level of training. It gives the user a sense of security in their daily work.

## Conclusions

1. We recommend to use the notation PR-E-(AUD_2_IT) for the complete algorithm.
2. Disregard the employment of action schemes such as SBAR or Sampler and instead adopt the PR-E-(AUD_2_IT)-algorithm instead as a comprehensive alternative algorithm.
3. Use the “PR-E-” as a chronologic guide to prepare for the treatment, but use (AUD_2_IT) as a chronological action scheme as well as a single-point handover-scheme.
4. There is no reason not to use the PR-E-(AUD_2_IT)-algorithm for other medical issues like in non-emergency outpatient departments or post-emergency-units. The process is the same, data are the same so the structure should be the same as well.

## Data Availability

The original contributions presented in the study are included in the article/Supplementary Material, further inquiries can be directed to the corresponding author.
